# Dispensing and Purchasing Antibiotics Without Prescription: A Cross-sectional Study Among Pharmacists and Patients in Beirut, Lebanon

**DOI:** 10.1177/00469580231167712

**Published:** 2023-04-13

**Authors:** Rasha Kakati, Sanaa Nakad Borrego, Rana Zareef, Johnny Atallah, Souha Farhat, Nahla Daye, Sandra Sadek, Marco Bardus

**Affiliations:** 1 American University of Beirut, Beirut, Lebanon; 2 University of Birmingham, Birmingham, UK

**Keywords:** antimicrobial resistance, antibiotic purchasing and dispensing, pharmacists, individual behavior, Lebanon

## Abstract

Antimicrobial resistance is a global public health issue, exacerbated by dispensing and purchasing antibiotics without a prescription, common in low- and middle-income countries, such as Lebanon. This study aimed to (1) describe behavioral patterns underpinning dispensing and purchasing antibiotics without a prescription among pharmacists and patients, (2) describe reasons for, and (3) attitudes toward these behaviors. A cross-sectional study targeted pharmacists and patients, respectively, identified through stratified random sampling and convenience sampling from all 12 Beirut quarters. Questionnaires assessed behavioral patterns, reasons for, and attitudes toward dispensing and purchasing antibiotics without prescription among the 2 samples. A total of 70 pharmacists and 178 patients were recruited. About a third (37%) of pharmacists supported dispensing antibiotics without a prescription, considering it acceptable; 43% of patients report getting antibiotics without a prescription. Reasons for distributing and purchasing antibiotics without prescription include financial costs associated with the drugs and convenience, coupled with inexistent law enforcement. Dispensing antibiotics without prescription was shared among a relatively high proportion of pharmacists and patients residing in Beirut. Dispensing antibiotics without prescription is common in Lebanon, where law enforcement needs to be stronger. National efforts, including anti-AMR campaigns and law enforcement, must be rapidly implemented to avoid the double disease burden, especially when old and new vaccines are available, and superbugs are making preventative public health efforts more difficult.

**What do we already know about this topic?**
Dispensing and purchasing antibiotics without prescription is an unlawful yet practiced behavior in low- and middle-income countries (LMICs); however, little is known about the views of pharmacists and patients.
**How does your research contribute to the field?**
This study compares the views of pharmacists and patients, providing a comprehensive picture of the phenomenon of dispensing/purchasing antibiotics without a prescription in an LMIC vexed by financial and economic crises.
**What are your research’s implications for theory, practice, or policy?**
This study shows that, albeit unlawful, dispensing and purchasing antibiotics without a prescription is a contemporary practice in Lebanon. To combat the phenomenon, the Ministry of Public Health and the Orders of Physicians and Pharmacists should introduce urgent interventions, including more rigid controls, law enforcement, and financial packages to support needy families.

## Introduction

According to the World Health Organization (WHO), antimicrobial resistance (AMR) is a global public health issue,^1^ as common infectious diseases cannot be effectively treated, resulting in prolonged illnesses, disabilities, and premature death. AMR also has significant repercussions on healthcare costs, as it can compromise the success of treatments.^1^ With increasing resistance rates discovered in every country, including Lebanon, some studies^2-4^ reported high rates of resistance to several types of bacteria, such as *Streptococcus pneumoniae*, *E. coli*, *Staphylococcus aureus*, *Acinetobacter*, and *Pseudomonas*.

While AMR might develop over time through genetic changes, the misuse and overuse of antimicrobial agents may accelerate this process.^5^ Some recent estimates from the Organization for Economic Co-operation and Development (OECD) show that inappropriate use of antimicrobials can reach up to 50%, and unneeded antibiotic prescriptions can range from 45% to 90% globally.^6^ For example, in Lebanon, a recent study showed that 65% of patients received a prescription for the wrong antibiotic, and the treatment was inappropriate in 79% of these cases.^7^ In 2016, the WHO published a global action plan on antimicrobial resistance to address this significant public health issue,^8^ still used today as a reference for developing national AMR plans. The global action plan is aligned with more recent strategic documents of the Food and Agriculture Organization of the United Nations (FAO)^9^ and the World Organization for Animal Health (WOAH), formerly known as OIE.^10^

The WHO plan acknowledged the role of healthcare professionals in minimizing the overuse and misuse of antibiotics. Pharmacists can play a central role in this effort, providing effective medication management for short- and long-term treatments. However, a recent systematic review highlighted the different approaches to AMR between high-income countries (HICs) and low- and middle-income countries (LMICs), the latter lagging due to limited resources to invest in training and enforce laws to counter antibiotic over-prescription.^11^ Where pharmacists are legally allowed to prescribe antibiotics, fast and reliable diagnostic tests can support them in correctly diagnosing common infections such as chlamydia or Lyme disease. In addition, they assess whether they can successfully treat a patient or whether the patient needs to be referred to another healthcare professional.

Inadequate knowledge about AMR and its consequences among physicians might drive inappropriate use,^12^ but patients increasingly self-medicate and rely on pharmacists’ dispensing antibiotics without prescription (DAWP); hence, the problem of overuse might be both from the supply and demand side. From the demand side, a recent study conducted in Lebanon showed that 22% of the surveyed participants self-prescribed antibiotics, while 7% followed the advice of non-medical individuals.^13^ From the supply side, pharmacists and healthcare professionals play an active role in dispensing antibiotics without following international guidelines^6^ or questioning patients’ demands. Some research conducted among Lebanese physicians^14-16^ showed that many prescribed inappropriate antibiotics. According to a recent systematic review on global access to antibiotics without prescription in community pharmacies,^17^ the rate of DAWP was 62%, which increased to 78% when a patient requested these drugs.^17^ Some research conducted in Lebanon confirmed that DAWP could range from 42% to 63%.^7,18^ Another recent systematic review on health practitioners’ knowledge, attitudes, and practice toward antibiotic prescribing and resistance in developing countries^19^ showed that AMR might not be linked only to practitioners’ knowledge level. Reasons for purchasing antibiotics without a prescription (PAWP) in low-income countries such as Lebanon included the need to save money and time,^13,18^ and low socioeconomic status,^20^ especially in disadvantaged neighborhoods, where people cannot afford medical visits, considering the fragmented and semi-private healthcare system.

Although previous studies reported a high prevalence of antibiotic overuse and misuse in Lebanon, little is known about the behavioral patterns and reasons behind DAWP and PAWP, respectively, from the point of view of pharmacists and patients. Specifically, this study aimed to (1) describe behavioral patterns related to DAWP and PAWP among pharmacists and patients, (2) describe reasons for, and (3) attitudes toward DAWP and PAWP.

## Methodology

### Design and Study Population

This cross-sectional study targets pharmacists, identified through stratified random sampling, and adult patients living in Beirut recruited through convenience sampling from public spaces in the same areas of the pharmacies. Before the interviews, a verbal consent form was obtained from all participants who received copies of the consent form. In addition, an oral consent form was deemed appropriate to minimize potential biases while ensuring participants’ confidentiality about the controversial issue investigated. This study was conducted between February 2019 and April 2019; the study protocol and procedures were approved by AUB’s Institutional Review Board (IRB) (ref no. SBS-2019-0061).

### Sampling

#### Pharmacists sample

A stratified random sampling framework was used to select pharmacies within each of the 12 quarters of the Municipal Beirut area, starting from a list on the Order of Pharmacists of Lebanon’s website.^21^ The pharmacies were filtered by governorate, district, and city of Beirut, excluding hospitals, para-pharmacies, or drug stores. The search yielded a total of 231 pharmacies, the total reference population. Using an online sample size calculator, assuming a confidence level of 95% (*z* = 0.95), a margin of error of 10% (*d* = 0.1), and maximum variability of 50% (*P* = .5) in the proportion of the outcome (ie, selling antibiotics without prescription), the minimum sample size was 69 pharmacies.

#### Patients sample

A convenience sampling approach was followed. Like for the pharmacists, we used a sample size calculator for an indefinite population with a confidence level of 95% (*z* = 0.95), a margin of error of 10% (*d* = 0.1), and a maximum variability of 50% (*P* = .5) in the outcome (PAWP). Based on these assumptions, at least 97 individuals were needed (approximately 8 patients for every 12 quarters of Beirut).

### Instruments and Procedures

Pharmacists completed a self-administered paper-and-pencil anonymous questionnaire (available in Arabic and English); patients were interviewed by research staff members using an anonymous questionnaire (in Arabic).

#### Pharmacists

The authors visited each selected pharmacy and spoke with the responsible head pharmacist (who could be a manager or owner), following an approved oral consent script. We approached the head pharmacist, who is responsible (both professionally and legally) for the conduct of their employees. If they agreed to participate, they received a copy of the consent form and were given 10 to 15 min to complete the survey. After the survey, the interviewer handed them an infographic presenting World Health Organization (WHO) recommendations concerning the pharmacist’s role in preventing AMR in Arabic. If the pharmacist did not agree to participate, they received the infographic, and the pharmacy was excluded from the study. Another pharmacy was selected from the list belonging to the same quarter of Beirut.

#### Patients

The team approached participants in crowded public spaces purposefully chosen to maximize variability in socioeconomic background. Crowded areas included busy streets, shopping malls, and grocery stores. Before starting the interview, oral consent was collected; if a patient did not want to participate, another person was approached. After the interview, the team member handed the participant an infographic containing WHO recommendations for preventing AMR.

All data were entered in a database developed using the Research Electronic Data Capture (REDCap),^22^ a secure web-based platform for building and managing online databases and surveys hosted on our institution servers.

### Measures

#### Pharmacists’ questionnaire

The pharmacist questionnaire included the following parts: (a) sociodemographic; (b) behaviors and patterns of antibiotic use; (c) reasons for dispensing antibiotics without prescription (DAWP); and (d) knowledge and attitudes toward DAWP. An English version of this questionnaire is provided in Supplemental Material 1. Based on a previous study,^20^ the sociodemographic section included the age, gender, and years of experience of the interviewed pharmacist. *Behavioral patterns of antibiotic dispensation* had questions related to antibiotic dispensation (eg, number of antibiotics dispensed, number of antibiotics dispensed without a prescription, etc.). In addition, this section included “I dispense antimicrobials without a prescription” (measured on a 5-point Likert scale). *Finally, reasons for DAWP* and *attitudes toward DAWP* had a series of yes/no questions adapted from a similar study conducted among community pharmacists in Saudi Arabia, as the questionnaire items were included in the original publication.^23^

#### Patients interview

This tool was aimed to assess behavioral patterns, reasons, and attitudes toward PAWP, and was based on similar studies conducted in various LMICs^24-26^ and Europe.^27^ The questionnaire encompassed 3 sections: (a) sociodemographic, (b) behaviors and patterns of antibiotic use, and (c) attitudes concerning access to antibiotics. An English version of this questionnaire is provided in Supplemental Material 1. *Sociodemographic information* included questions on citizenship, age, gender, area of residence where they purchased medications, level of education, employment status, and perceived socioeconomic status. *Behavioral patterns of antibiotic use* included questions aimed at recalling when and where patients bought antibiotics and whether this happened with a prescription; this section had the question “Do you usually obtain a prescription before purchasing an antibiotic?” which was used as our indicator of PAWP support. *Reasons for PAPW* and *attitudes concerning access to antibiotics*: included three 5-point Likert scale items developed for this study (“Leftover antibiotics are good to keep at home in case they may be needed later”; “It’s good to be able to get antibiotics from relatives or friends without having to see a doctor”; “It’s good to be able to buy antibiotics from a pharmacy without having to see a doctor”).

### Data Management and Analyses

The datasets were exported from REDCap into SPSS for analysis. Descriptive statistics were performed to describe sociodemographic characteristics, behavioral patterns of DAWP and PAWP, and reasons and attitudes toward DAWP and PAWP. For example, in the pharmacist dataset, the responses to the statement “I dispense antimicrobials without a prescription” were used to create a variable “DAWP support,” dichotomized as follows: strongly agree and agree = I support DAWP; neutral, disagree, strongly disagree = I do not support DAWP—we made the conservative assumption that pharmacists who answered neutrally were not performing the behavior.

The results reported the study objectives. Independent sample *t*-tests (for continuous variables) and chi-square tests (for categorical variables) were used to assess the associations between DAWP support, PAWP support, and the other factors. In addition, the proportions of DAWP and PAWP were compared qualitatively and using chi-square tests.

## Results

### Recruitment Results

Of 104 approached pharmacists, 70 agreed to participate and completed the questionnaire (response rate: 67%). Among 349 patients, 178 agreed to participate in the study (response rate: 51%). The 2 samples covered the 12 quarters of Municipal Beirut, similarly, as shown in Table 1 below. The characteristics of the sample of pharmacists are presented in Table 2, and those of patients are shown in Table 3 below. Briefly, the sample of pharmacists included respondents who were, on average, 41 years old (SD = 10.5; range = 23-64), primarily male (53%), who had been practicing for 14 years, on average (SD = 8.2; range = 2-33). The sample of patients mainly included female respondents (59%), predominantly Lebanese (96%), with an average age of 33 years (SD = 13.4; range: 18-77), who achieved at least a bachelor-level education (69%), currently employed (54%), with a moderate perceived socioeconomic status (52%), and with health insurance (71%).

**Table 1.** Distribution of the Samples of Pharmacists and Citizens Across the Quarters of Municipal Beirut.

Quarter, n (%)	Pharmacists (n = 70)	Citizens (n = 178)
Marfaa	1 (1.4)	9 (5.1)
Zuqaq El Blat	3 (4.3)	10 (5.6)
Achrafieh	13 (18.6)	26 (14.6)
Dar El Mreisseh	1 (1.4)	1 (0.6)
Mousaitbeh	14 (20.0)	17 (9.6)
Rmeil	1 (1.4)	6 (3.4)
Mina El Hosn	1 (1.4)	1 (0.6)
Bachoura	2 (2.9)	9 (5.1)
Saifi	2 (2.9)	9 (5.1)
Ras Beirut	13 (18.6)	33 (18.5)
Mazraa	18 (25.7)	40 (22.5)
Medawar	1 (1.4)	5 (2.8)
Other	0 (0.0)	12 (6.7)
**Total**	**70 (100.0)**	**178 (100.0)**

**Table 2.** Sociodemographic Characteristics and Behavioral Patterns of Antibiotic Dispensation Among Pharmacists (n = 70).

Characteristics	Total sample of pharmacists (n = 70)	DAWP support^a^ (yes, n = 26)	DAWP support^a^ (no, n = 44)	*P*-value^b^
Gender, n (%)				0.627
Male	37 (52.9)	15 (57.7)	22 (50.0)	
Female	32 (45.7)	11 (42.3)	21 (47.7)	
Missing	1 (1.4)	0 (0.0)	1 (2.3)	
Age, M (SD)	41.3 (10.5)	40.3 (10.2)	41.8 (10.8)	0.572
Years of professional experience, M (SD)	14.3 (8.2)	13.8 (8.2)	14.7 (8.3)	0.657
How many antibiotics do you dispense per day?, n (%)				0.426
Less than 10	36 (51.4)	11 (42.3)	25 (56.8)	
Between 11 and 30	27 (38.6)	12 (46.2)	15 (34.1)	
More than 31	6 (8.6)	3 (11.5)	3 (6.8)	
Missing	1 (1.4)	0 (0.0)	1 (2.3)	
Number of DAWP (How many antibiotics without a prescription do you dispense per day?), n (%)				0.001
Less than 10	56 (80.0)	16 (61.5)	40 (90.9)	
Between 11 and 30	10 (14.3)	9 (34.6)	1 (2.3)	
More than 31	3 (4.3)	1 (3.8)	2 (4.5)	
Missing	1 (1.4)	0 (0.0)	1 (2.3)	
Frequency of DAWP (How many times people ask for antibiotics without a prescription per day?), n (%)				0.077
Many times per day	40 (57.1)	20 (76.9)	20 (45.5)	
Once a day	7 (10.0)	1 (3.8)	6 (13.6)	
2-3 times per week	13 (18.6)	4 (15.4)	9 (20.5)	
Less than 2-3 times per week	9 (12.9)	1 (3.8)	8 (18.2)	
Missing	1 (1.4)	0 (0.0)	1 (2.3)	
Do you prescribe antibiotics to infants?, n (%)				0.020
Yes	12 (17.1)	18 (69.2)	40 (90.9)	
No	58 (82.9)	8 (30.8)	4 (9.1)	
Do you prescribe antibiotics to children?, n (%)				0.041
Yes	40 (57.1)	5 (19.2)	19 (43.2)	
No	30 (42.9)	21 (80.8)	25 (56.8)	
Do you prescribe antibiotics to older adults?, n (%)				0.011
Yes	43 (61.4)	5 (19.2)	22 (50.0)	
No	27 (38.6)	21 (80.8)	22 (50.0)	
I dispense antibiotics without prescription, n (%)^a^				<0.001
Strongly disagree	9 (12.9)	(0.0)	9 (20.5)	
Disagree	9 (12.9)	(0.0)	9 (20.5)	
Neutral	26 (37.1)	(0.0)	26 (59.1)	
Agree	23 (32.9)	23 (88.5)	(0.0)	
Strongly agree	3 (4.3)	3 (11.5)	0 (0.0)	

DAW*P* = Dispensing without prescription.

a DAWP support is based on the variable “I dispense antibiotics without prescription” (strongly agree and agree = yes; strongly disagree, disagree, and neutral = no).

b *P*-value of the chi-square test (Fisher’s exact test) or *t*-test comparing DAWP support groups.

**Table 3.** Sociodemographic Characteristics and Behavioral Patterns of Antibiotic Purchasing Among Citizens (n = 178).

Characteristics	Sample of citizens (n = 178)	PAWP support^a^ (no, n = 54)	PAWP support^a^ (yes, n = 122)	*P*-value^b^
Gender, n (%)				.321
Male	70 (39.3)	25 (46.3)	44 (36.1)	
Female	105 (59.0)	29 (53.7)	75 (61.5)	
missing	3 (1.7)	0 (0.0)	3 (2.4)	
Age, M (SD)	33.0 (13.4)	32.0 (13.7)	33.5 (13.4)	.501
Citizenship, n (%)				1.000
Lebanese	171 (96.1)	52 (96.3)	117 (95.9)	
Other	7 (3.9	2 (3.7)	5 (4.1)	
Education, n (%)				.196
Less than a bachelor	56 (31.5)	13 (24.1)	43 (35.2)	
Bachelor or more	122 (68.5)	41 (75.9)	79 (64.8)	
Employment status, n (%)				.463
Unemployed	67 (37.6)	24 (44.4)	42 (34.4)	
Employed	96 (53.9)	26 (48.1)	69 (56.6)	
Other	15 (8.4)	4 (7.4)	11 (9.0)	
Perceived socioeconomic status, n (%)				.911
Low	28 (15.7)	8 (14.8)	20 (16.4)	
Moderate	93 (52.2)	28 (51.9)	64 (52.5)	
High	49 (27.5)	15 (27.8)	34 (27.9)	
I don’t know	8 (4.5)	3 (5.6)	4 (3.3)	
Do you have medical insurance?, n (%)				.359
Yes	126 (70.8)	35 (64.8)	90 (73.8)	
No	49 (27.7)	17 (31.5)	31 (25.4)	
Missing	3 (1.7)	2 (3.7)	1 (0.8)	
When was the last time you purchased antibiotics?, n (%)				.316
Today	2 (1.1)	1 (1.9)	1 (0.8)	
Last week	11 (6.2)	3 (5.6)	8 (6.6)	
Last month	41 (23.0)	9 (16.7)	30 (24.6)	
In the last 6 months	66 (37.1)	20 (37.0)	46 (37.7)	
In the last year	23 (12.9)	6 (11.1)	17 (13.9)	
More than a year ago	17 (9.6)	5 (9.3)	12 (9.8)	
I cannot remember	18 (10.1)	10 (18.5)	8 (6.6)	
On that occasion, did you get a prescription from a doctor?, n (%)				<.001
Yes	90 (50.6)	9 (20.5)	81 (66.4)	
No	69 (38.8)	35 (79.5)	32 (26.2)	
Not applicable/missing	19 (10.7)	10 (18.5)	9 (7.4)	
Frequency of PAWP (How frequently do you purchase antibiotics that were not prescribed to you by a physician?), n (%)				<.001
Always	6 (3.4)	6 (11.1)	0 (0.0)	
Often	30 (16.9)	21 (38.9)	7 (5.7)	
Sometimes	42 (23.6)	14 (25.9)	28 (22.9)	
Rarely	60 (33.7)	11 (20.4)	49 (40.2)	
Never	39 (21.9)	2 (3.7)	37 (30.3)	
Missing	1 (0.6)	0 (0.0)	1 (0.8)	

PAW*P* = purchasing antibiotics without prescription.

a PAWP support is based on the variable “Do you usually obtain a prescription before purchasing an antibiotic?” (yes/no).

b *P*-value of the chi-square test (Fisher’s exact test) or *t*-test comparing PAWP support groups.

### Behavioral Patterns Related to DAWP and PAWP

#### Pharmacists

Most respondents admitted to dispensing less than 10 antibiotics without a prescription (56/70, 80%), stating that patients requested these many times daily (40/70, 57%). The majority declared they *did not*: prescribe antibiotics to infants (58/70, 83%); however, many admitted prescribing antibiotics to children (40/70, 57%) or the elderly (43/70, 61%). Twenty-six respondents out of 70 (37%) agreed or strongly agreed with the statement “I dispense antimicrobials without a prescription,” and 18 disagreed or strongly disagreed (18/70, 26%); the same amount was “neutral” (26/70, 37%). Based on these data, DAWP support included 26 pharmacists in favor (37%) and 44 against it (63%). DAWP support was significantly associated with the number of antibiotics dispensed without prescription (*Χ*^2^(2) = 13.298, *P* = .001), prescribing antibiotics to infants (*Χ*^2^(1) = 5.407, *P* = .020), children (*Χ*^2^(1) = 4.161, *P* = .041), and to the elderly (*Χ*^2^(1) = 6.530, *P* = .011).

#### Patients

Most respondents (160/178, 90%) recalled purchasing antibiotics in the last 6 months or less. Many got a prescription from a doctor (90/160, 56%), but many did not (69/160, 43%). In most cases, patients declared they usually obtained a prescription before purchasing antibiotics (122/178, 69%) and rarely or never purchased antibiotics without a prescription (99/178, 56%).

### Reasons for DAWP and PAWP

**Figure 1.**
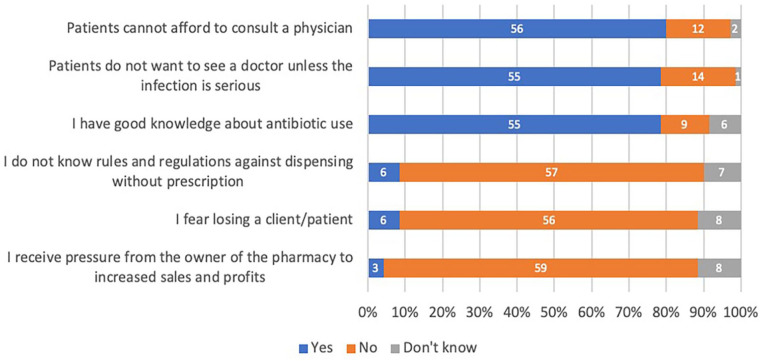
Reasons for DAWP among pharmacists (n = 70).

The reasons for DAWP and PAWP are respectively displayed in Figure 1 and Table 4 below.

**Table 4.** Reasons for PAWP Among Citizens (n = 178).

Reasons	n	%	% of cases
Previous successful experiences	74	24.3	54.8
Doctors tend to prescribe the same antibiotic	43	14.1	31.9
Saving time	70	23.0	51.9
Saving money	46	15.1	34.1
Easier	58	19.0	43.0
Other reasons	14	4.6	10.4
**Total**	**305**	**100.0**	**225.9**

*Note.* The reasons were added as multiple choice, so the counts do not add up to the sample size.

#### Pharmacists

Several pharmacists interviewed believed that they dispense antibiotics without a prescription because they feel patients cannot afford the price of a consultation with a physician (56/70, 80%) or do not want to see a doctor unless the infection is severe (55/70, 79%). Also, they believed they had good knowledge about antibiotic use (55/70, 79%). However, few admitted that the reason for dispensing without prescription was due to the ignorance of rules and regulations against it (6/70, 9%), or to the fear of losing a patient (6/70, 9%), or the perceived pressure to increase sales (3/70, 4%).

#### Patients

The most frequent reasons for purchasing an antibiotic without a prescription were past behavior (55%), or because it saves time (52%), it was easier (43%), it allows to keep money (34%), or because the doctors tend to prescribe the same antibiotic (32%).

### Attitudes Towards DAWP and PAWP

**Figure 2.**
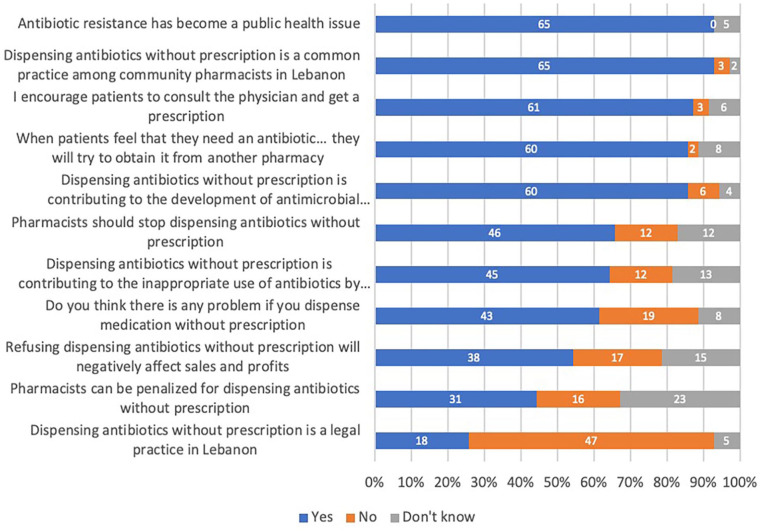
Knowledge and attitudes toward DAWP among pharmacists.

**Figure 3.**
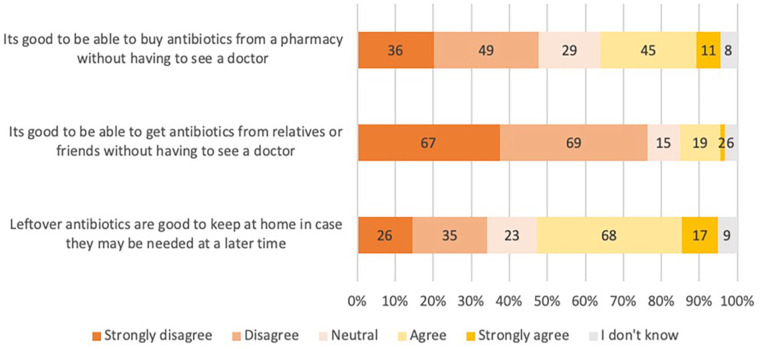
Attitudes toward purchasing antibiotics without a prescription among patients.

The attitudes toward DAWP and PAWP are summarized in Figures 2 and 3 below.

#### Pharmacists

Most interviewed pharmacists acknowledged that AMR had become a public health issue (65/70, 93%) and that DAWP contributes to AMR (60/70, 86%). However, many believe that DAWP is a common practice in the country (65/70, 93%). While most declared to encourage patients to get a prescription from a physician (61/70, 87%), about two-thirds of the sample believed that pharmacists should stop DAWP (46/70, 66%) because it is contributing to the inappropriate use of antibiotics by patients (45/70, 64%). It is a significant problem (43/70, 61%). About half of the sample believed that refusing DAWP would negatively affect their business (38/70, 54%). More than a quarter of the interviewees thought that DAWP is a legal practice (18/70, 26%).

#### Patients

Most patients believed it is not good to get antibiotics from relatives or friends without seeing a doctor (136/178, 76%). About half of the sample believed it is not good to buy antibiotics from a pharmacy without seeing a doctor (85/178, 48%). Leftover antibiotics are good to keep at home if needed later (85/178, 48%).

### Comparing DAWP and PAWP: Behavioral Estimates

The proportion of pharmacists answering the option “less than 2 to 3 times/week” in the question “How many times per day are people asking to dispense antibiotics without a prescription?” was compared to the proportion of patients answering “Never” in the question “How frequently do you purchase antibiotics that were not prescribed to you by a physician.” As shown in Table 5 below, the proportions of DAWP and PAWP were similar (the chi-square test was not significant). These were also aligned with the proportions of DAWP support (37.1%) and PAWP support (30.3%). When patients were asked about their latest antibiotic purchase, about half reported getting a prescription (90/159, 57%).

**Table 5.** Comparison Between Pharmacists’ and Citizens’ Estimates.

Estimate	Pharmacists (n = 70)	Citizens (n = 178)	*P*-value
Frequency of DAWP (less than 2-3 times/week)^a^ versus frequency of PAWP (never)^b^	9 (12.9)	34 (19.1)	.242
DAWP support (yes), n (%) versus PAWP support (yes), n (%)	26 (37.1%)	54 (30.3)	.302
When you last purchased an antibiotic, did you get a prescription from a doctor? (yes), n (%)		90/159 (56.6)	

a Frequency of DAWP is based on the question “How many times per day are people asking to dispense antibiotic without prescription?” (Less than 2-3 times/week).

b Frequency of PAWP is based on the question “How frequently do you purchase antibiotics that were not prescribed to you by a physician” (Never); DAWP support: I support dispensing antibiotics without prescription versus I do not support; PAWP support: “Do you usually obtain a prescription before purchasing an antibiotic?” (yes).

## Discussion

### Behavioral Patterns Related to DAWP and PAWP

This study described patterns, reasons behind, and attitudes toward DAWP and PAWP, comparing the points of view of patients and pharmacists. These were similar, confirming that DAWP and PAWP are present in Lebanese society. Many pharmacists believe that AMR is a public health issue and that DAWP contributes to AMR, in line with existing review evidence.^17,19^ Pharmacists play an essential role in antimicrobial resistance prevention, and their function can be formalized through participation in antimicrobial stewardship programs.^28,29^ Pharmacists are the most accessible healthcare professionals and are fully competent in all aspects of medicine. They serve as communicators and educators on healthy behaviors and infection prevention, providing expertise and advice on AMR. Pharmacists in other countries promote focused education and training on AMR and advocate optimizing antimicrobial use, antimicrobial stewardship, and mitigating AMR in all resource settings.^30^ Especially in countries with low law enforcement and a prevalence of illegal practices, pharmacists can be true gatekeepers of information and influence consumer behaviors.^9,10^

The frequency of DAWP reported by pharmacists (many times a day: 55.7%) was very close to that reported among pharmacists in affluent areas of Beirut (50%) said in a previous study by Farah et al.^20^ This is also aligned with the global review evidence, estimating the prevalence of DAWP to be around 62% without a patient request and 78% with a patient request^17^; in this study, DAWP support as a proxy indicator for prevalence (37%) was also similar to the estimated 32% prevalence in Farah et al’s^20^ study. The frequency of DAWP reported by pharmacists was identical to that of PAWP reported by patients (30%). This estimate aligns with other studies among Lebanese patients (ranging from 22%^13^ to 42%^18^). The differences might be due to the sampling techniques employed or the different way questions were asked, relying on self-report or observations. Our estimates show that DAWP in Lebanon is higher than in neighboring countries such as Saudi Arabia. Similar studies found a prevalence of around 13% following law enforcement^31^ and much higher proportions among those unaware of its illegality.^23^ Yet, it is much lower than in other low- and middle-income countries such as Africa (eg, Zambia^32^ and Ghana^25^) and Asia (eg, Indonesia^24^ and India^26^). Future studies should find a better and more objective way to estimate DAWP without relying directly on pharmacists’ viewpoints. For example, some studies employed “mystery shopping” or “patient simulation” methodologies to simulate realistic scenarios whereby researchers pretend to be patients and ask for antibiotics without a prescription.^33,34^ While these techniques raise ethical concerns about using deception and obtaining deferred consent from pharmacists, they might provide a more accurate picture of underreported illegal behaviors such as DAWP.

### Reasons for DAWP and PAWP

The reasons for DAWP and PAWP reported in this study are aligned with the literature: the main reasons for not requesting a prescription must deal with financial issues from the patient’s side, as reported in other previous studies conducted in (LMICs),^24-26^ Lebanon,^20^ and Saudi Arabia.^23^ In addition, due to the fragmented and privatized nature of the healthcare system, patients tend to avoid visiting physicians unless necessary.^20,23,25^ In this study, socioeconomic status was not significantly associated with PAWP, but this might be due to the way patients were approached and to the fact that a pretty homogeneous sample was recruited (ie, relatively well-educated and with moderate or high socioeconomic backgrounds) compared to other studies which looked at the differences in sociodemographic areas where pharmacies were based^20^ and in other cross-sectional studies done among the general population living in Lebanon, education and socioeconomic status played an important role in determining preventive behaviors such as cancer screening,^35^ health information seeking,^36^ or access to healthcare services.^37^

### Attitudes Towards DAWP and PAWP

The attitudes toward DAWP and PAWP were mixed, with about two-thirds of pharmacists believing that DAWP is a problem they should stop. However, most of the interviewees acknowledged that DAWP is a common practice. In addition, while most pharmacists acknowledged the existence of regulations against DAWP, many believed it was legal. Also, many patients thought it was good to purchase antibiotics without a prescription or keep leftovers at home so they would not need to buy new ones. While these findings align with the literature,^7,9,11,14,19^ it is worrisome to see how DAWP and PAWP are embedded and accepted in the culture.

### Limitations and Way Forward

This study investigated the behavioral patterns related to DAWP and PAWP among pharmacists and patients of different areas of Beirut, describing the reasons for and attitudes toward these behaviors. While it was not aimed at assessing the prevalence of DAWP and PAWP in the country, the study provides evidence suggesting that some pharmacists still sell antibiotics without a prescription, and patients request them. The findings of this study should be interpreted with some limitations. First, the sampling techniques and the relatively small sample size limit the generalizability of the results to other parts of Lebanon. Due to the nature of DAWP and social desirability bias, pharmacists might have underreported DAWP. We tried to minimize this by collecting patient information, but patients also provided self-reported data, which is prone to under or over-reporting. Future studies should try to assess the actual behavior through direct observation of DAWP and PAWP among patients and pharmacists using techniques that minimize social desirability bias (eg, mystery shopping or “social experiments”).

## Conclusion

This study shows that dispensing and purchasing antibiotics without a prescription is common in Lebanon and accepted by pharmacists and patients. While pharmacists acknowledge that antibiotic resistance is a public health issue and understand their role in reducing it, some still sell them without a prescription. At the same time, patients are also prone to request antibiotics without a prescription. With the crippling economy and financial crisis that Lebanon has been facing since the end of 2019, reducing this phenomenon might not be easy. Yet, it would be an essential step toward healthcare for all. The Ministry of Public Health to the Orders of Physicians and Pharmacists should introduce urgent interventions, including more rigid controls and law enforcement of a recently implemented law against DAWP. In parallel, governments should consider pushing the agenda of national health coverage to include antibiotics so that more and more segments of the population can afford to purchase medications without worrying about paying for medical consultations. In addition, stringent surveillance and enforcement of a recent law are needed to address this problem from the supply side; from the demand side, more accessible, cheaper access to medications should be provided so that patients could be more likely to obtain prescriptions for all antibiotics they need. A sustainable financial support package should be delivered to families most in need. Once this system is in place, public health communication campaigns about AMR could be implemented to raise awareness about this critical public health issue.

## Supplemental Material

sj-docx-1-inq-10.1177_00469580231167712 – Supplemental material for Dispensing and Purchasing Antibiotics Without Prescription: A Cross-sectional Study Among Pharmacists and Patients in Beirut, Lebanon Click here for additional data file. Supplemental material, sj-docx-1-inq-10.1177_00469580231167712 for Dispensing and Purchasing Antibiotics Without Prescription: A Cross-sectional Study Among Pharmacists and Patients in Beirut, Lebanon by Rasha Kakati, Sanaa Nakad Borrego, Rana Zareef, Johnny Atallah, Souha Farhat, Nahla Daye, Sandra Sadek and Marco Bardus in INQUIRY: The Journal of Health Care Organization, Provision, and Financing
