# Supplementary material for: Dispensing and Purchasing Antibiotics Without Prescription: A Cross-sectional Study Among Pharmacists and Patients in Beirut, Lebanon
Source: Inquiry. 2023 Apr 13;60:00469580231167712. doi: 10.1177/00469580231167712 (PMC10102940; doi:10.1177/00469580231167712)
Supplement: sj-docx-1-inq-10.1177_00469580231167712 – Supplemental material for Dispensing and Purchasing Antibiotics Without Prescription: A Cross-sectional Study Among Pharmacists and Patients in Beirut, Lebanon [file sj-docx-1-inq-10.1177_00469580231167712.docx]

# Supplemental Material 1

## Questionnaire for Pharmacists

**Sociodemographic information (derived from Farah et al. 2015)**

| Age | Number of years |
| --- | --- |
| Sex | Male  Female |
| How many years of experience as a pharmacist do you have? | Number of years |
| How many antibiotics are dispensed per day at the pharmacy? | Less than 10  Between 11 and 30  More than 31 |
| How many antibiotics are dispensed per day without prescription? | Less than 10  Between 11 and 30  More than 31 |
| How many times per day are people asking to dispense antibiotic without prescription? | Many times a day  Once a day  2-3 times per week  Less than 2-3 times per week |
| Do you prescribe antibiotics to infants? | Yes / No |
| Do you prescribe antibiotics to children? | Yes / No |
| Do you prescribe antibiotics to elderly? | Yes / No |
| I dispense antimicrobials without a prescription | Strongly disagree  Disagree  Neutral  Agree  Strongly agree |

**Reasons for dispensing antibiotics without prescription (adapted from Hadi et al. 2016)**

| **Options** | **Answers** |
| --- | --- |
| I have good knowledge about antibiotic use | Yes/No |
| Patients do not want to see a doctor unless the infection is serious | Yes/No |
| I receive pressure from the owner of the pharmacy to increased sales and profits | Yes/No |
| Patients cannot afford to consult a physician | Yes/No |
| I fear losing a client/patient | Yes/No |
| I do not know rules and regulations against dispensing without prescription | Yes/No |

**Knowledge and attitudes towards dispensing antibiotics without prescription (adapted from Hadi et al. 2016)**

| Dispensing antibiotics without prescription is a legal practice in Lebanon | Yes | No | Don’t know |
| --- | --- | --- | --- |
| Dispensing antibiotics without prescription is a common practice among community pharmacists in Lebanon | Yes | No | Don’t know |
| Do you think there is any problem if you dispense medication without prescription | Yes | No | Don’t know |
| Dispensing antibiotics without prescription is contributing to the development of antimicrobial resistance | Yes | No | Don’t know |
| Antibiotic resistance has become a public health issue | Yes | No | Don’t know |
| Dispensing antibiotics without prescription is contributing to the inappropriate use of antibiotics by patients | Yes | No | Don’t know |
| Pharmacists can be penalized for dispensing antibiotics without prescription | Yes | No | Don’t know |
| Pharmacists should stop dispensing antibiotics without prescription | Yes | No | Don’t know |
| I encourage patients to consult the physician and get a prescription | Yes | No | Don’t know |
| When patients feel that they need an antibiotic, if not dispensed, they will try to obtain it from another pharmacy | Yes | No | Don’t know |
| Refusing dispensing antibiotics without prescription will negatively affect sales and profits | Yes | No | Don’t know |

## Questionnaire for Citizens

*Behavioral patterns of antibiotic use*

| When was the last time you purchased antibiotics?  (If cannot remember, skip to next section) | Today  Last week  Last month  In the last 6 months  In the last year  More than a year ago  Cannot remember | |  |
| --- | --- | --- | --- |
| On that occasion, did you get a prescription for it from a doctor?  (If not, skip to 6) | Yes  No | |  |
| Do you usually obtain a prescription before purchasing an antibiotic? | | Yes  No | |
| How frequently do you purchase antibiotics that were not prescribed to you by a physician?  (If never, skip to 14) | | Always  Often  Sometimes  Rarely  Never | |
| If you purchase without prescription, what are your reasons? | | Previous successful experiences  Doctors tend to prescribe the same antibiotic  Saving time  Saving money  Easier  Other… | |

Attitudes concerning access to antibiotics

To what extent do you agree with the following statements? Please use a scale of 5, where 5 means Strongly Agree and 1 means Strongly disagree.

| Leftover antibiotics are good to keep at home in case they may be needed later | Strongly Agree  Agree  Neither  Disagree  Strongly disagree  I don’t know |
| --- | --- |
| It’s good to be able to get antibiotics from relatives or friends without having to see a doctor | Strongly Agree  Agree  Neither  Disagree  Strongly disagree  I don’t know |
| It’s good to be able to buy antibiotics from a pharmacy without having to see a doctor | Strongly Agree  Agree  Neither  Disagree  Strongly disagree  I don’t know |

Sociodemographic information: Last, a few questions about you

| Citizenship | Lebanese  Other | |
| --- | --- | --- |
| Age in years |  | |
| Gender | Male  Female | |
| Can you confirm your district of residence? | Marfaa  Zuqaq El Blat  Achrafieh  Dar El Mreisseh  Mousaitbeh  Rmeil | Mina El Hosn  Bachoura  Saifi  Ras Beirut  Mazraa  Medawar |
| What is your highest degree or level of education completed | No schooling completed  12^th^ grade or less, no diploma/qualifications  High school graduate with diploma/qualifications  Some college credit, no degree  Technical/vocational training or associate degree  Bachelor’s degree  Master’s/professional degree  Doctorate degree | |
| Are you currently employed? | Unemployed  Employed  Other | |
| Compared to other people your age, how well-off do you think you/your family is? | A lot poorer than most  A little poorer than most  About the same as most  A little richer than most  A lot richer than most  I don’t know | |
| Do you have a medical insurance? | Yes  No | |
